# Apport du scanner multi-coupe dans les anomalies des arcs et de l'arche aortique chez l'enfant

**DOI:** 10.11604/pamj.2019.33.312.17831

**Published:** 2019-08-20

**Authors:** Badr Slioui, Achraf Zaimi, Latifa Chat

**Affiliations:** 1 Service d'Imagerie Médicale 4ème Hôpital Militaire Dakhla, Maroc; 2 Service de Cardiologie 4ème Hôpital Militaire Dakhla, Maroc; 3 Service d'Imagerie Médicale, Hôpital Pédiatrique Rabat, Maroc

**Keywords:** Enfant, malformations cardio-vasculaires, arcs aortiques, coarctation de l’aorte, interruption et hypoplasie de l’aorte, angioscanner thoracique, Child, cardio-vascular malformations, aortic arches, coarctation of the aorta, interruption and hypoplasia of the aorta, CT angiogram

## Abstract

Les malformations congénitales des arcs aortiques correspondent à un groupe hétérogène de pathologies liées à des anomalies de développement des arcs branchiaux primitifs pendant la vie intra-utérine. La coarctation de l'aorte est une malformation vasculaire congénitale fréquente qui correspond à un rétrécissement congénital de l'isthme aortique, segment de l'aorte compris entre l'artère sous-clavière gauche et le canal artériel. L'interruption de l'arche aortique est considérée par certains auteurs comme une forme extrême de la coarctation de l'aorte, caractérisée par une discontinuité entre l'aorte ascendante et descendante. Ces anomalies s'intègrent dans la majorité des cas dans le cadre de malformations cardiaque dont ils sont indissociables. L'angioscanner thoracique joue un rôle incontournable dans l'exploration de ces anomalies, leurs bilans préopératoires et leurs suivis à long terme. Notre travail s'inscrit dans le cadre d'une étude rétrospective portant sur 42 patients explorés tous par angioscanner thoracique à la suite de la découverte d'une cardiopathie sur l'examen échocardiographique. L'angiographie a été réalisée dans 6 cas. L'âge moyen est de 2 ans et 6 mois avec des extrêmes allant de 6 jours à 14 ans. Nous avons noté une prédominance masculine avec un sexe ratio de 1,6. Les principales pathologies rencontrées étaient: la coarctation aortique: 18 cas; les hypoplasies de l'arche aortique: 8 cas; les interruptions de l'arche aortique: 7 cas; les anomalies des arcs aortiques: 9 cas. Certaines de ces anomalies étaient associées. Les anomalies extra-cardiaques associées aux cardiopathies congénitales sont relativement fréquentes; le scanner multibarrete permet une bonne analyse des voies afférentes et efférentes au cœur. Grace à une exploration tri-dimensionnelle et multiplanaire satisfaisante, la tomodensitometrie (TDM) permet en complément de l'échocardiographie d'établir un bilan pré-thérapeutique des principales pathologies malformatives surtout pour la détection des anomalies vasculaires extra-cardiaques associées. Elle tend à supplanter l'angiographie dans de nombreuses pathologies malformatives et ce pour plusieurs raisons: il est moins invasif; il offre une image 3D d'excellente qualité utile au chirurgien; il établie le diagnostic anatomique, apprécie la compression trachéale et fait le bilan d'éventuelles malformations associées; il guide le traitement chirurgical.

## Abstract (en)

Congenital malformations of the aortic arches are a heterogeneous group of diseases associated with developmental disorders of the primitive branchial arches during fetal life. The coarctation of the aorta is a common congenital vascular malformation which is a congenital narrowing of the aortic isthmus, a segment of the aorta between the left subclavian artery and the ductus arteriosus. The interruption of the aortic arch is considered by some authors as an extreme coarctation of the aorta, characterized by discontinuity between ascending and descending aorta. These abnormalities are integrated, in most cases, in the context of cardiac malformations from which they are indivisible. CT angiogram plays an essential role in the examination of these abnormalities, their preoperative assessment and their follow-up in the long term. We conducted a retrospective study of 42 patients undergoing CT angiogram following the detection of heart disease on echocardiography. CT angiogram was performed in 6 cases. The average age of patients was 2 years, ranging from 6 days to 14 years; a male predominance was reported with a sex ratio of 1,6. The main diseases were: coarctation of the aorta: 18 cases; hypoplasies of the aortic arch: 8 cases; interruptions of the aortic arch: 7 cases; abnormalities of the aortic arches: 9 cases. Some of these abnormalities were associated. Extracardiac abnormalities associated with congenital heart diseases are relatively frequent; multislice scanner allows for good analysis of the cardiac afferent and efferent pathways. CT complements echocardiogram for pre-treatment assessment of the main malformative diseases, especially for the detection of the associated extra-cardiac vascular abnormalities, thanks to its satisfactory tridimensional multiplanar exploration. It tends to supplant angiography in many pathological malformations for several reasons: it is less invasive; it provides high-resolution 3D images useful to surgeons; it established the anatomical diagnosis, assesses tracheal compression and any associated malformation; it guides surgical treatment.

## Introduction

Les cardiopathies congénitales correspondent à un groupe de pathologies dû à un développement anormal des structures cardiaques pendant la vie intra-utérine, responsable de malformations plus ou moins complexes du cœur et des gros vaisseaux [1]. L'incidence de ces pathologies, y compris les anomalies les plus banales, est de 75-81/1000 naissances vivantes; tandis que l'incidence des anomalies nécessitant une prise en charge post-natale est de 2,5-3,0/1000 naissances vivantes [2,3]. Les malformations des arcs aortiques correspondent à un groupe hétérogène de pathologies liées à des anomalies de développement des arcs aortiques touchant la crosse aortique, ses branches ou encore l'artère pulmonaire [4]. L'interruption de l'arche aortique est considérée comme une forme extrême de coarctation de l'aorte, caractérisée par une discontinuité entre l'aorte ascendante et descendante [5]. La connaissance des substratums anatomiques et embryologiques permet une meilleure compréhension de ses aberrations, qui sont souvent associées parfois dans des formes complexes. D'importantes avancées techniques ont permises une amélioration de la prise en charge de ces malformations, tant sur le plan diagnostic que thérapeutiques, du diagnostic prénatale jusqu'à l'âge adulte. Le couple échocardiographie et angiographie étaient les examens de première intention pour le diagnostic en cardiologie pédiatrique. L'échographie permet de poser le diagnostic de certitude dans la majorité des cas; cependant elle comporte certaines insuffisances tenant moins à la qualité d'image dans cette population très échogène qu'aux limites propres de la technique, à savoir l'étude des structures extracardiaques. L'angiographie est la technique complémentaire usuelle; elle-même comportant des insuffisances liées au caractère irradiant, invasif et surtout une étude limite à la lumière n'incluant pas l'environnement juxta-vasculaire. Les autres techniques d'imagerie trouvent là tous leurs intérêts, en complément de l'échographie. L'imagerie 3D, IRM et surtout le scanner multi-barrette, permettent une excellente étude des vaisseaux extracardiaques dans le cadre du bilan pré-thérapeutique; ils sont également utiles pour l'évaluation post-opératoires notamment l'étude des montages chirurgicaux qui sont parfois complexes. A première vue, l'IRM semble la technique idéale en raison de son caractère non invasif notamment chez les petits enfants et nouveau nés, mais sa résolution spatiale est inférieure à celle du scanner et le temps d'examen plus long [6-8]. L'angioscanner multi-barrette, grâce à des acquisitions rapides et une adaptation des protocoles pour limiter l'irradiation représente un réel progrès dans l'exploration des cardiopathies congénitales et permet aux radiologues et aux cliniciens d'avoir accès, de façon non invasive, à une imagerie tridimensionnelle du cœur et des gros vaisseaux [6-9]. Les objectifs de ce travail sont: détailler les modalités techniques qui permettent de réaliser un angioscanner thoracique chez l'enfant; préciser l'apport de l'angioscanner dans l'exploration des anomalies des arcs aortiques et de l'arche de l'aorte à travers une série de 42 enfants explorés dans le cadre d'une cardiopathie congénitale; évaluer l'intérêt du scanner multi-coupe par rapport à l'échographie trans-thoracique et l'angiographie dans le diagnostic, le bilan préopératoire et le suivi à long terme.

## Méthodes

### Sélection des patients

**Critères d'inclusion:** âge: inférieur ou égal à 16 ans. Les patients présentant une cardiopathie congénitale explorés par une échocardiographie-doppler et angioscanner multi barrette.

**Critères d'exclusion:** patients n'ayant pas été explorés par TDM.

### Technique

Etude rétrospective, 42 patients explorés tous par échocardiographie et angioTDM thoracique. L'angiographie a été réalisée dans 6 cas. Les examens ont été réalisés par deux appareils de scanner; 16 détecteurs et 32 détecteurs (64 coupes); acquisition hélicoïdale depuis les troncs supra-aortiques (base du cou) jusqu'au diaphragme puis reconstructions multiplanaires (MPR), Maximum Intensity Projection (MIP), et reconstructions 3D en rendu de volume.

### Grille d'interprétation

L'indication de l'angioscanner a été portée avec accord entre les cardiologues, radiologues et le chirurgien cardiaque lorsqu'une hypothèse chirurgicale entrait en compte. Ont été analysés; morphologie cardiaque; situs, connexions physiologiques, CIA, CIV, péricarde, valves; morphologie extracardiaque; aorte (coarctation ou dilatation), arcs aortiques, artères pulmonaires, veines pulmonaires ou systémiques, artères coronaires. Biométrie des cavités cardiaques et des vaisseaux extra-cardiaques; rapport ventriculaire droit (VD)/ventriculaire gauche (VG) mesuré selon 2 plans; 4 cavités et petit axe; aorte, tronc de l'artère pulmonaire, artères pulmonaires droite et gauche; analyse des voies aériennes et du parenchyme pulmonaire, confrontation des résultats de l'écho-cœur et de la TDM avec les données chirurgicales.

## Résultats

### Analyse épidémiologique

**Age:** l'âge moyen est de 2 ans et 6 mois avec des extrêmes allant de 6 jours à 14 ans (Tableau 1). 27 patients, soit 64% avaient un âge inférieur à deux ans.

**Tableau 1:** caractéristiques sociodémographiques, différents signes cliniques et répartition des pathologies

Tranches d’âge	Nombre de malades
[0-2 ans]	27
[2-4 ans]	4
[4-6 ans]	2
[6-8 ans]	2
[8-10 ans]	2
[10-12 ans]	2
[12-14ans]	3
**Sexe**	
Masculin	26
Féminin	16
**Signes cliniques**	
Cyanose	27
Détresse respiratoire	9
Dyspnée	7
RSP	5
Broncho-pneumopathie à répétions	3
**Pathologies**	
Coarctation aortique	18
Hypoplasie de l’arche aortique	8
Interruption de l’arche aortique	7
Anomalies des arcs aortiques	9
Découverte fortuite	1

**Sexe:** nous avons noté une prédominance masculine, le sexe ratio H/F est de 1,6 (Tableau 1).

**Clinique:** les signes cliniques rencontrés étaient variables selon le type de cardiopathie. Les principaux signes rencontrés étaient: la cyanose (37%), la détresse respiratoire (22%) et la dyspnée (17%) (Tableau 1).

**Répartition des pathologies:** (Tableau 1) certaines de ces anomalies étaient souvent associées.

### Analyse par pathologie

**Figure 1:**
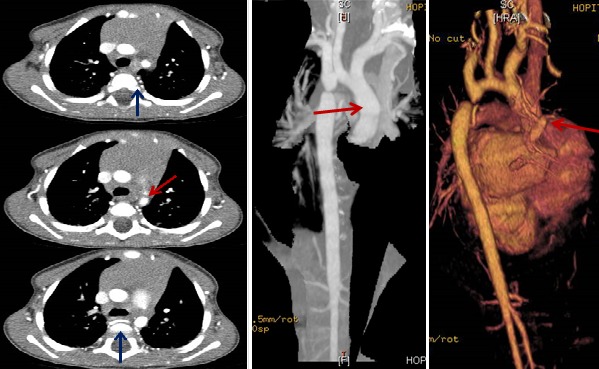
coarctation pré-ductale (isthme)

**La coarctation de l'aorte:** 18 cas ont été recensés. Les extrêmes d'âge: 7 jours-14 ans. Le diagnostic a été fait avant l'âge de 1 mois chez 9 patients (50%). Une prédominance masculine a été notée à 67%. Clinique: 80% des malades présentait une détresse respiratoire, parfois associés à des signes de défaillance cardiaque. Examens d'imagerie réalisés et leurs résultats: échocardiographie et TDM: dans tous les cas; angiographie: 4 cas; radiographie pulmonaire et ECG: tous les cas Tableau 2. Iconographie: Figure 1. Le canal artériel était persistant dans 64% des cas avec un diamètre allant de 2mm à 6mm, pour une moyenne de 3,4mm. Les circulations collatérales étaient présentes dans 42% des cas, souvent via les artères péri vertébrales. Les anomalies associées: HTAP: 44%; hypoplasie de l'arche: 28%; CIV: 16%; artéria lusoria: 6%; transposition des gros vaisseaux: 6%.

**Tableau 2:** résultats pour la coarctation

			Echocardiographie	Angioscanner
siège	Ductale		2	2
	Pré-ductale		8	9
	Post-ductale		-	7
Forme	Localisé	Serré	-	14
		Moyenne	-	2
	Tubulaire		-	2
Aorte d’amant hypoplasique			-	5
Aorte d’aval dilaté			-	2
Canal artériel			8	11
HTAP, AP dilatée			8	8
Circulations collatérales			-	7

**Figure 2:**
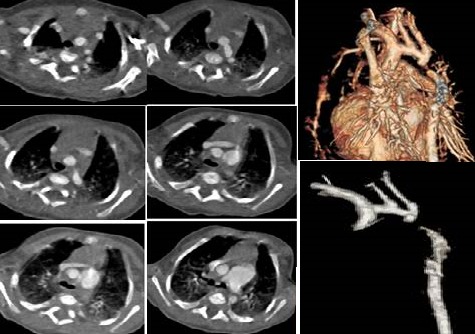
interruption de l'arche aortique type A

**Figure 3:**
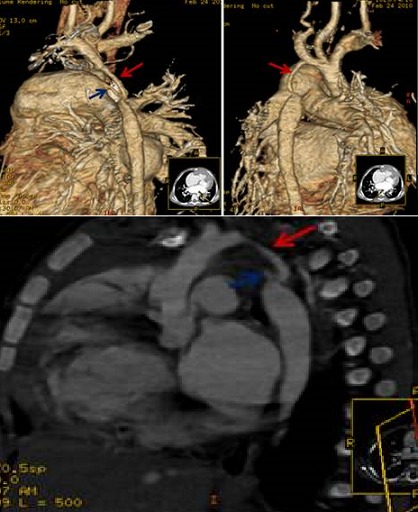
hypolasie de l'arche aortique basse

**Interruption et hypoplasie de l'arche aortique:** 15 cas ont été recensés dont un est une association des deux. Les extrêmes d'âge: 2 jours-10 ans. Le diagnostic a été fait avant l'âge de 1mois chez 6 patients (40%). Une prédominance masculine a été notée à 67%. Clinique: détresse respiratoire (50%), signes de défaillance cardiaque. Examens d'imagerie réalisés leurs résultats: échocardiographie et TDM: dans tous les cas; angiographie: 2 cas; radiographie pulmonaire et ECG: tous les cas Tableau 3. Iconographie: Figure 2, Figure 3. Canal artériel perméable: 40% des cas avec un diamètre allant de 2mm à 9mm, pour une moyenne de 3,4mm. Le tronc de l'AP était dilaté dans 13% des cas. Les circulations collatérales étaient présentes dans 20% des cas, souvent via les artères péri vertébrales. Les anomalies associées: HTAP: 61%; hypoplasie de l'arche: 5%; CIV: 4%; bicuspidie: 9%; tronc commun des carotides: 4%; KingKing: 4%; TGV: 4%; anomalie du retour veineux: 9%.

**Tableau 3:** résultats pour les interruptions et hypoplasies de l’arche

		Echocardiographie	Angioscanner
Interruption	Type A	3	5
	Type B	-	2
	Type C	-	0
Hypoplasie	haute	-	4
	basse	1	4
Relai par TAP		-	12
Relai par TAP via canal artériel		-	6
Circulation collatérale péri-vertébrale		-	3

**Figure 4:**
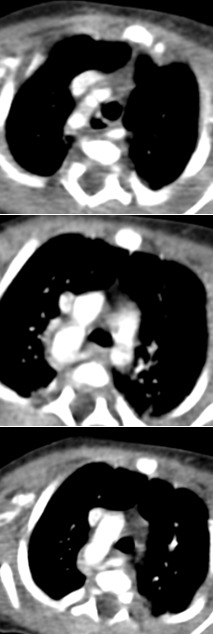
arc aortique droit

**Figure 5:**
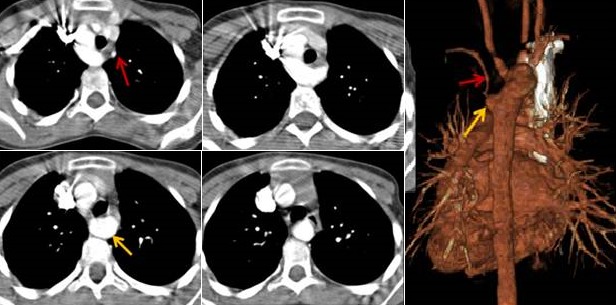
double arc aortique

**Figure 6:**
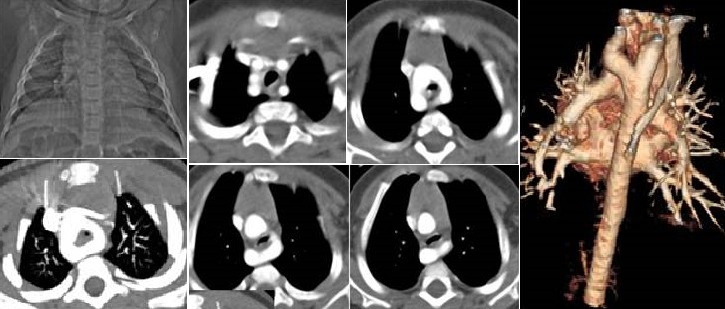
anomalie de NEUHAUSSER

**Figure 7:**
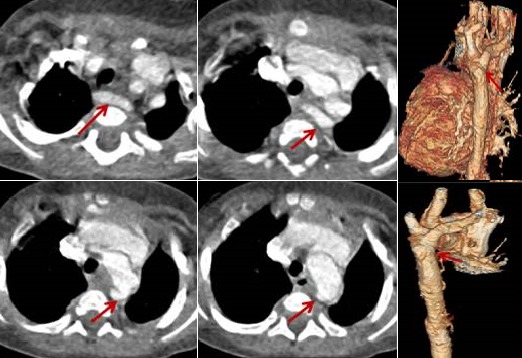
artéria lusoria

**Anomalies des arcs aortiques:** 9 cas ont été recensés; les extrêmes d'âge: 11 jours-14 ans; le diagnostic a été fait avant l'âge de 1 mois chez 2 patients (20%); une prédominance masculine a été notée à 52%; clinique: détresse respiratoire (80%), signes de défaillance cardiaque. Différents types rencontrés: arc aortique droit: 4 cas Figure 4; Neuhausser: 2 cas Figure 5; double arc aortique: 2 cas (Figure 6); arétria lusoria: 1 cas (Figure 7). Les anomalies associées: anomalie du retour veineux: 33%; absence de TVI: 16%; CIV: 17%; canal artériel perméable: 17%; Artéria lusoria: 17%.

## Discussion

### Malformations étudiées

#### La coarctation aortique

La coarctation de l'aorte est une malformation vasculaire congénitale fréquente qui correspond à un rétrécissement congénital de l'isthme aortique, segment de l'aorte compris entre l'artère sous-clavière gauche et le canal artériel. Elle représente 4 à 6/10000 naissances, 6 à 8% de l'ensemble des cardiopathies congénitales et serait plus fréquente chez les filles. Les syndromes associés sont rares, à l'exception du syndrome de Turner où elle est retrouvée dans 15 à 25% des cas [6, 10, 11, 12]. Elle représente 26% des cardiopathies rencontrées dans notre étude.

**Physiopathologie:** la coarctation isthmique résulte d'un défaut de développement de l'aorte horizontale distale, et plus particulièrement de l'isthme aortique. La coarctation peut être simple ou associée à une hypoplasie de l'arche aortique ou à d'autres malformations intracardiaques.

#### Diagnostic et évaluation préopératoire

**Diagnostic anténatal:** fait dans plus de 40% des cas et permet de réduire la mortalité néonatale préopératoire.

#### Diagnostic post natal

**Echocardiographie doppler:** examen à demander en première intention en cas de suspicion de coarctation. Il permet de visualiser directement un rétrécissement de l'isthme aortique, de préciser la forme anatomique de la coarctation et d'évaluer le retentissement de coarctation sur le ventricule gauche.

**Apport du scanner:** le scanner précise le type de coarctation (en avant ou en arrière du canal artériel), évalue le degré de coarctation et la taille de la crosse aortique et peut détecter la présence de thrombus au niveau de la coarctation. Il permet une évaluation préopératoire tridimensionnelle, qui peut aider au choix de la voie d'abord chirurgicale (sternotomie médiane ou thoracotomie latérale). Il permet aussi l'évaluation du résultat chirurgical et détection des complications: recoarctation, hypoplasie résiduelle de l'arche aortique, faux anévrysme [12-15]. Dans notre étude, le scanner était aussi performant que l'échocardiographie dans le diagnostic positif de la coarctation. Il était plus performant que l'échographie dans la détection des anomalies vasculaires extra-cardiaques associées: anomalies de l'arche, circulation collatérale et il était moins performant dans la détection des anomalies intracardiaques: bicuspidie.

**Radiographie thoracique:** souvent normale ou parfois érosions costales (bord inférieur des côtes moyennes au niveau du segment postérieur).

**Interruption de l'arche:** l'interruption de l'arche aortique est considérée par certains auteurs comme une forme extrême de coarctation de l'aorte, caractérisée par une discontinuité entre l'aorte ascendante et descendante. La TDM permet grâce aux reconstructions multi planaires et 3D d'effectuer un bilan lésionnel complet et de montrer la topographie exacte de l'interruption par rapport aux troncs supra-aortiques [16]. Trois cas d'interruptions aortiques diagnostiquées par TDM ont été recensés.

**Anomalies des arcs aortiques:** ce sont des malformations congénitales fréquentes liées à des anomalies de développement des arcs branchiaux primitifs. Elles représentent 1% des anomalies congénitales cardio-vasculaires [13].

##### Anomalies des 4^èmes^ arcs aortiques

**Double arc aortique:** il résulte de la persistance des deux 4^ème^s arcs; il s'agit de la forme la plus fréquente des anneaux vasculaires aortiques symptomatiques. L'aorte ascendante se prolonge par deux arcs de part et d'autre de la trachée, se joignant en arrière pour former l'aorte descendante. La TDM précise l'anatomie détaillée du double arc comme était le cas chez un de nos patients: dimensions et position de chaque arc, dominance de l'un par rapport à l'autre, rapports entre l'anneau vasculaire et la trachée, degré de compression trachéale et œsophagienne, position des troncs supra-aortiques sur les arcs [5, 13].

**Artère sous clavière de trajet aberrant:** l'imagerie montre une naissance distale de l'artère sous clavière droite à la jonction de l'arche et de l'aorte descendante. Il peut également s'agir d'une artère sous clavière gauche de trajet aberrant sur un arc [5, 16] droit. Dans 80% des cas, elle est retro-œsophagienne, intertrachéo-œsophagienne dans 15% des cas et pré trachéale dans 5% des cas. Nous avons noté un cas d'artère sous clavière aberrante retro-œsophagienne.

**Arc droit avec disposition en miroir des troncs supra aortiques et aorte descendante a droite du rachis:** l'imagerie doit préciser ces rapports anatomiques, signaler et décrire le diverticule de Kommerell s'il est présent et rechercher des signes de compression trachéale [16].

**Anomalie de Neuhausen:** elle représente 15 à 20% des anomalies symptomatiques des arcs aortiques. Un stridor, des infections pulmonaires récurrentes et une dysphagie sont fréquents. La trachée et/ou l'œsophage sont comprimés par un anneau vasculoligamentaire représenté par un arc droit d'un côté et le ligament artériel à gauche [16].

**Anomalie de longueur du 4^ème^ arc aortique: aorte « kingking »:** crosse aortique anormalement longue et sinueuse sans anomalie de position des troncs supra-aortiques. La TDM a permis de faire le diagnostic de cette anomalie chez un patient, prise à l'échographie pour une coarctation. Le scanner a par ailleurs révélé chez ce patient d'autres anomalies associées non visualisées à l'échocardiographie: RVPA et double système collecteur [16].

##### Anomalies des 6^èmes^ arcs aortiques

**Agénésie d'une artère pulmonaire:** Le poumon homolatéral peut être absent ou hypoplasique; anomalie asymptomatique dans 30% des cas [16].

**Artère pulmonaire gauche rétro-trachéale:** l'artère pulmonaire gauche se développe à partir de l'artère pulmonaire droite, passe entre la trachée et l'œsophage et provoque un anneau vasculaire compressif [16].

##### Autres anomalies

**Arche aortique cervicale:** résulte de la persistance du 3^ème^ arc aortique, de sorte que l'arche aortique est en position haute à la jonction cervico-thoracique. L'aorte cervicale réalise une masse battante expansive du creux sus-sternal.

**Artère sous clavière gauche isolée:** cette anomalie résulte de la double interruption du 4^ème^ arc aortique gauche entre l'artère sous clavière gauche et l'origine de l'aorte descendante d'une part, et l'artère carotide primitive et l'artère sous clavière gauche, d'autre part. L'arche aortique est à droite.

### Persistance du 5^ème^ arc aortique

**Origine anormale du tronc artériel brachiocéphalique:** l'imagerie montre le trajet de la gauche vers la droite de ce tronc et son rapport avec la face antérieure de la trachée [16].

### Anomalies associées

**Aux coarctations de l'aorte:** la coarctation de l'aorte s'intègre dans le cadre d'un syndrome de Turner dans 15% des cas [10-12]. Dans 60% des cas, elle peut être associée à d'autres malformations, à savoir: l'hypoplasie de l'arche aortique; CIV; bicuspidie aortique; anomalie mitrale, syndrome de Shone. Dans notre série, nous avons noté une association à l'hypoplasie de l'arche aortique dans 28% des cas, à une CIV dans 16% des cas, à une artèria lusoria dans 6% des cas et à une transposition des gros vaisseaux dans 6% des cas.

**Aux hypoplasies et interruptions de l'arche aortique:** les anomalies les plus décrites dans la littérature sont [16]: la persistance du canal artériel; fenêtre aorto-pulmonaire; transposition des gros vaisseaux; Truncus. Dans notre série, nous avons noté des associations suivantes: hypoplasie de l'arche; CIV; bicuspidie; tronc commun des carotides; kingking; transposition des gros vaisseaux; anomalies du retour veineux systémique.

**Aux anomalies des arcs aortiques:** on a noté en plus des associations entre les différents types d'anomalie des arcs aortiques, une association à d'autres malformations tel que: anomalies du retour veineux; CIV; canal artériel perméable.

## Conclusion

Grâce à sa résolution spatiale infra millimétrique, le scanner multi-détecteurs tend à supplanter l'angiographie dans le bilan des malformations des gros vaisseaux et ce pour plusieurs raisons: il est moins invasif que l'angiographie; il offre une imagerie 3D d'excellente qualité utile au chirurgien; il établit le diagnostic anatomique, apprécie la compression trachéale, et fait le bilan d'éventuelles malformations associées; il guide le traitement chirurgical. En pratique, la prise en charge d'un enfant ayant une cardiopathie congénitale comporte une double évaluation: pour les anomalies intra-cardiaques: écho-cardiographie; pour les anomalies extra-cardiaques: TDM et IRM; pour la mesure des préssions: cathétérisme cardiaque. L'imagerie 3D de haute précision qu'offre la TDM multicoupe est une révolution dans l'exploration anatomique des malformations cardiaques ce qui fait de la TDM outil diagnostic et décisionnel.

### État des connaissances actuelles sur le sujet

- Malformations vasculaires congénitales rares;
- Association avec des malformations cardiaques;
- Exploration basée sur le couple échocardiographie et angioscanner thoracique et cathétérisme cardiaque.

### Contribution de notre étude à la connaissance

- Apporte des donnés épidémiologiques de la pathologiques selon notre série;
- Détailler le rôle de l'angioscanner aux différentes étapes de la prise en charge;
- Illustrations iconographiques.

## Conflits d’intérêts

Les auteurs ne déclarent aucun conflit d'intérêts.
